# A fur plucking model to study herpes simplex virus reactivation and recurrent disease

**DOI:** 10.1128/msphere.00783-23

**Published:** 2024-10-09

**Authors:** Drake T. Philip, Nigel M. Goins, Helen M. Lazear

**Affiliations:** 1 Department of Microbiology and Immunology, University of North Carolina at Chapel Hill, Chapel Hill, North Carolina, USA; The University of Arizona, Tucson, Arizona, USA

**Keywords:** herpes simplex virus, latency, reactivation, skin infection, dorsal root ganglia

## Abstract

Herpes simplex viruses (HSV-1 and HSV-2) most commonly cause ulcerative epithelial lesions (cold sores and genital herpes). Importantly, HSV establishes life-long persistent (latent) infection in peripheral neurons. Reactivation from latency produces recurrent epithelial lesions, which constitute the greatest burden of HSV disease in people. The mechanisms that regulate latency and reactivation remain incompletely understood, in part due to limitations in the animal models available for studying HSV reactivation. We have developed a simple and tractable model to induce HSV-1 and HSV-2 reactivation from latency to cause recurrent skin disease. We infected C57BL/6 mice with HSV-1 (strains NS, F, SC16, 17syn+) or HSV-2 (strain 333) on flank skin depilated by manual plucking. After at least 35 days post-infection (dpi), we replucked the fur from the infected flank and observed recurrent lesions in the same dermatome as the primary infection. We detected HSV DNA in dermatome skin through 4 days post-replucking and observed viral antigen and reporter signal in skin lesions by histology, consistent with viral replication following reactivation. In addition to C57BL/6 mice, we were able to produce reactivation in Balb/c and SKH-1 mice. We found that shaving the ipsilateral flank or plucking the contralateral flank did not induce recurrent skin lesions, suggesting that fur plucking is a specific stimulus that induces HSV reactivation. Furthermore, we were able to induce multiple rounds of plucking-induced recurrent disease, providing a model to investigate the lifelong nature of HSV infection. This new model provides a tractable system for studying pathogenic mechanisms of and therapeutic interventions against HSV reactivation and recurrent disease.

**IMPORTANCE:**

Herpes simplex viruses (HSV-1 and HSV-2) have infected over half of the US adult population to cause a lifelong, persistent infection; however, our understanding of the mechanisms that govern HSV reactivation and recurrent disease is incomplete. This is in part due to limitations in the animal models used to study recurrent disease, which are laborious and inefficient in mice. To address this technical gap, we developed a mouse model in which fur plucking after flank skin infection is sufficient to induce episodes of HSV reactivation and recurrent disease. Our work provides a model for the field to investigate the pathogenic mechanisms of HSV and immune responses during recurrent disease and provides an opportunity to investigate the neurobiology of HSV infection.

## INTRODUCTION

HSV-1 and HSV-2 are alphaherpesviruses with double-stranded DNA genomes of ~150 kb. HSV is a significant human pathogen, with 54% of US adults being seropositive for HSV-1 and 16% seropositive for HSV-2 (1). HSV is transmitted through close contact at cutaneous and mucosal barriers, including the skin, cornea, and genitals, and causes lifelong persistent infections by establishing latency in peripheral neuron cell bodies, primarily sensory neurons (2). During both acute and recurrent disease episodes, HSV primarily causes orofacial and genital lesions; however, in rare cases, HSV can cause encephalitis, primarily in children or in individuals with underlying immunodeficiencies (3–7). Historically, HSV-1 was considered to cause orofacial lesions and HSV-2 to cause genital lesions. However, either virus can infect at either site, and HSV-1 is responsible for a growing proportion of genital herpes cases (1, 8–12). The prevalence of both HSV-1 and HSV-2 infection at cutaneous and mucosal barriers highlights the need to understand the pathogenic mechanisms of HSV and the neurobiology that drives latency and reactivation at these sites. Furthermore, the greatest burden of HSV disease results from the ability of HSV to reactivate and cause recurrent disease throughout the lifetime of an infected person, highlighting the need to better understand mechanisms of HSV reactivation and recurrent disease.

Common animal models to study HSV reactivation use corneal inoculation, in mice or rabbits, where recurrent disease is challenging to evaluate, and assessment of reactivation is dependent upon detecting viral genomes in eye swabs (13). Rabbits have been used to study corneal HSV-1 reactivation because they support spontaneous and induced HSV-1 reactivation, depending upon the strain of HSV-1 used (13–17). Guinea pigs are used to study both ocular and genital HSV reactivation and disease because of their susceptibility to HSV-1 and HSV-2, their ability to support spontaneous reactivation, and straightforward detection of disease signs (13, 18–22). However, genetic and immunologic tools for guinea pigs and rabbits are far more limited than for mice. Most studies using mouse models of HSV reactivation rely on induction stimuli because spontaneous reactivation does not occur efficiently in mice (13, 23–25). Stimuli used to induce HSV reactivation in mice include ultraviolet light (26–29), hyperthermia (26, 30–32), stress (33, 34), and hormone treatment (35), but these can have variable efficiency and have limited tractability because of their reliance upon the ocular disease model. The lack of tractable mouse models to study HSV reactivation has been a barrier in the field, limiting progress in understanding the viral and host mechanisms that govern HSV latency, reactivation, and recurrent disease. Our work studying HSV pathogenesis in a mouse skin infection model (36) led us to develop an improved system to study HSV reactivation and recurrent disease.

## RESULTS

### Plucking reactivates HSV to cause recurrent skin disease in mice

**Fig 1:**
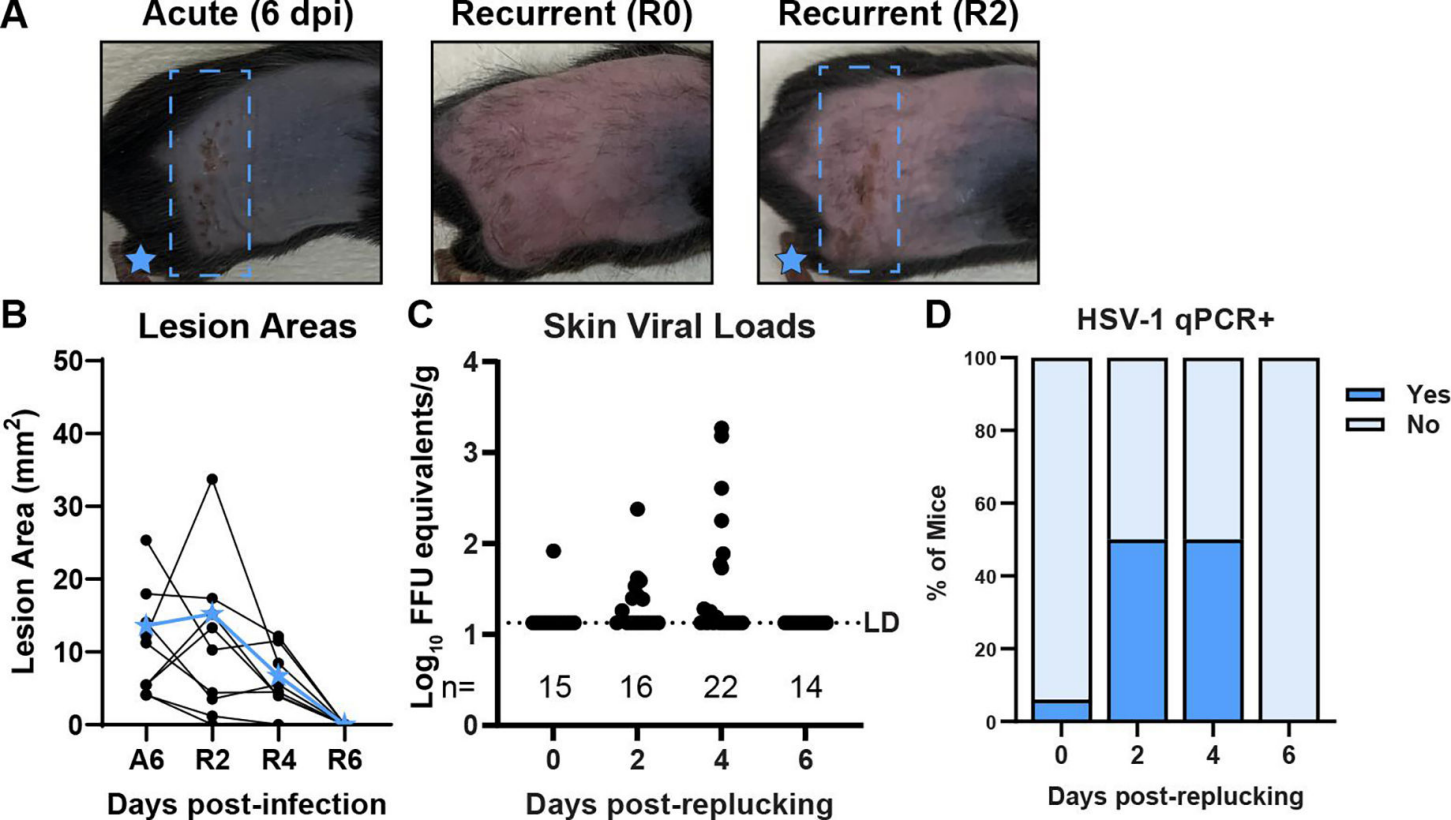
Plucking induces recurrent skin lesions in mice previously infected with HSV-1. WT (C57BL/6) mice were depilated by plucking 1 day prior to inoculation with 10^6^ FFU of HSV-1 strain NS. After 35 days, mice were replucked and evaluated for recurrent skin disease. Blue boxes denote the dermatome area of interest. (**A**) Representative serial photos of the same mouse during acute disease (6 dpi), immediately post-replucking (R0), and 2 days post-replucking (R2). (**B**) Skin lesion areas for individual mice were measured using ImageJ from photographs during acute disease (A6) or 2, 4, or 6 days post-replucking. Blue stars indicate the lesion areas of the mouse depicted in (**A**). (**C**) Dermatome skin was harvested at the indicated times after replucking, and viral loads were measured by qPCR. The limit of detection and number of mice per group are indicated. (**D**) Frequency of HSV-1 positive skin by qPCR.

We used a mouse flank skin infection model (37–46), which we previously optimized to reduce operator-to-operator and mouse-to-mouse variability (36). In brief, 1 day prior to infection, we depilated mice by manual fur plucking, then punctured the skin with an allergy needle, and applied viral inoculum over this abrasion (36). In this model, the virus replicates in the skin at the inoculation site, infects innervating peripheral neurons, traffics to the neuron cell bodies in the dorsal root ganglia (DRG), and then returns to the entire dermatome innervated by that DRG, producing a skin lesion. We measure the area of dermatome skin lesions from photographs and measure viral loads in the skin lesions by qPCR. We previously found that depilation by plucking had no effect on skin lesion area or viral loads compared to depilation by shaving plus chemical depilation with Nair cream (36). However, based on prior studies that used tape stripping-induced reactivation with an ear pinna infection model (47), we asked whether fur plucking was sufficient to induce HSV reactivation and recurrent disease. We infected male and female C57BL/6 (wild-type) mice with 1 × 10^6^ focus-forming units (FFU) of HSV-1 strain NS, evaluated their acute skin disease 6 days post-infection (dpi), then allowed the infection to resolve, the fur to regrow, and the virus to establish latency. Five weeks after the infection, we replucked the ipsilateral flank and evaluated skin lesions through 6 days post-reactivation. While no dermatome lesions were evident immediately upon replucking (recurrent day 0, R0), consistent with immune-mediated clearance of the acute lesion and establishment of viral latency, by 2 days post-reactivation (R2), mice exhibited skin lesions in the same dermatome as their acute disease. These recurrent lesions decreased by 4 days (R4) and resolved by 6 days post-reactivation (R6) (Fig. 1A and B).

To determine whether recurrent lesions corresponded to HSV-1 infection in dermatome skin, we infected male and female wild-type mice with HSV-1, replucked 35 dpi, collected dermatome skin from R0 to R6, and measured HSV-1 DNA by qPCR (Fig. 1C and D). There was no significant difference in the median skin viral loads between R2 and R4 (*P* = 0.63), although the maximum viral load of the positive samples increased at R4 (3.27 log_10_ FFU equivalents at R4 compared to 2.38 at R2) (Fig. 1C). While only 1 of 15 mice had detectable viral DNA in the skin at R0, by R2, we detected HSV-1 DNA in the skin of 8 of 16 mice. At R4, the frequency of HSV-1-positive animals was similar to that of R2 (11 of 22) (Fig. 1D). All mice cleared HSV-1 from the skin by R6 (Fig. 1C and D). The relatively low viral loads we observed are consistent with other models of HSV reactivation (14, 19, 28, 29) and with the presence of adaptive immune responses generated in response to HSV skin infection (37, 39, 41, 44, 46). The observation that the frequency of recurrent lesions was greater than the frequency of viral DNA detection is consistent with work from our lab and others, indicating that HSV disease is at least in part immune mediated and not directly correlated with viral burden (29, 36, 37, 48). Altogether, our results suggest that the stimulus of fur plucking induced HSV-1 to reactivate from latently infected DRG and traffic to dermatome skin, where it replicated and produced lesions.

**Fig 2:**
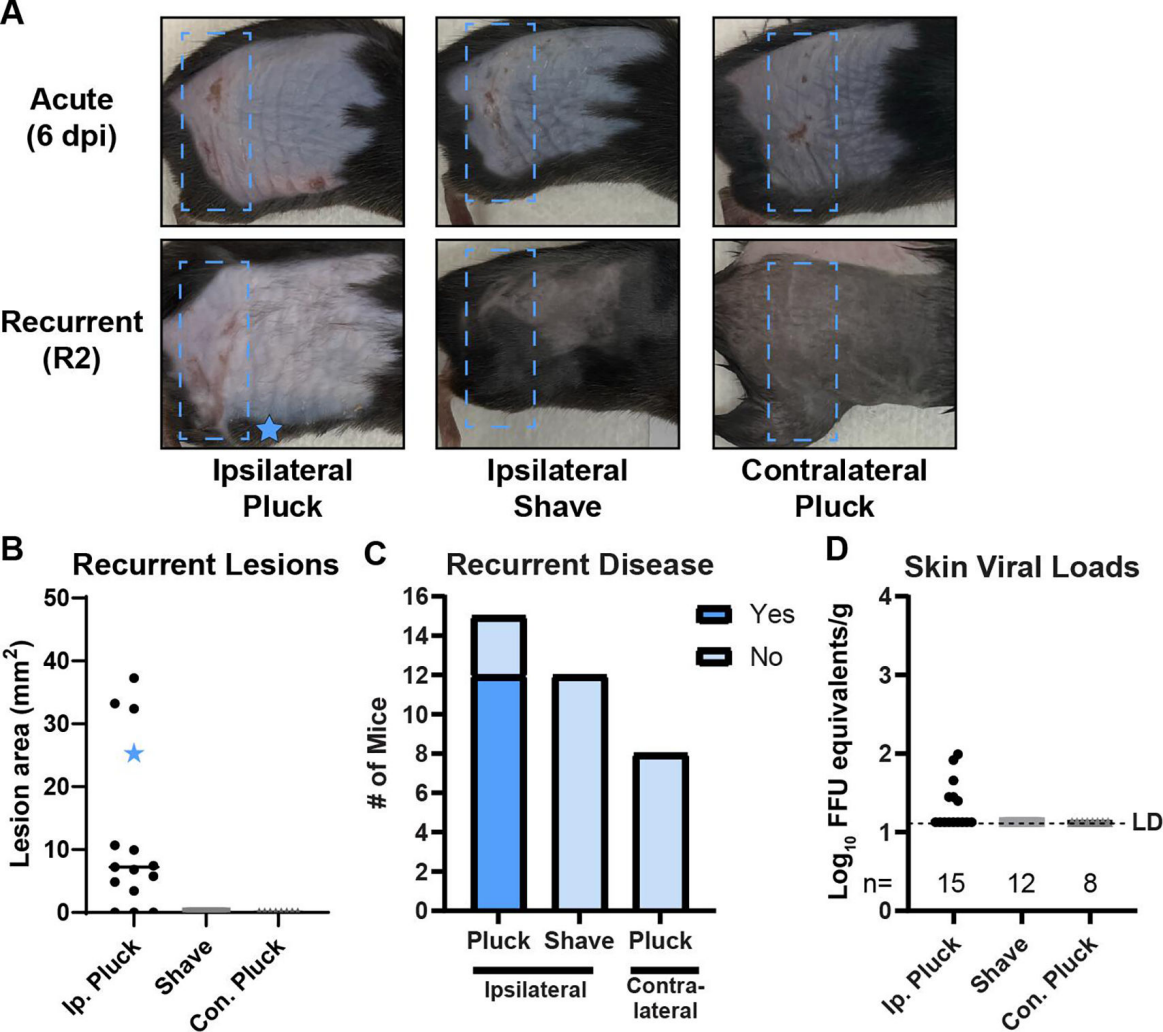
Ipsilateral shaving or contralateral plucking is not sufficient to cause HSV-1 recurrent skin disease. WT mice were depilated by plucking 1 day prior to inoculation with 10^6^ FFU of HSV-1 strain NS. After 35 days, mice were either replucked on their ipsilateral flank, shaved on their ipsilateral flank, or plucked on their contralateral flank (plus shaved on their ipsilateral flank to allow skin lesions to be observed). Mice were then evaluated at R2 for skin lesions and viral loads. (**A**) Representative serial photos of mice during acute disease (6 dpi) and recurrent disease (R2). Blue boxes denote the dermatome area of interest. Blue star indicates the lesion area of the mouse depicted in (**A**). (**B**) Skin lesion areas were measured from photographs at R2 using ImageJ. (**C**) Frequency of recurrent disease observed at R2. (**D**) Dermatome skin was harvested at R2, and viral loads were measured by qPCR. The limit of detection and number of mice per group are indicated.

We next asked whether fur plucking *per se* induced HSV-1 reactivation or whether reactivation might be induced by the stress of handling/manipulating the mice or temperature changes due to fur removal. To investigate this, we infected male and female wild-type mice with HSV-1 and allowed the virus to establish latency. At 35 dpi, we depilated the ipsilateral flank by plucking or by shaving with clippers (Fig. 2A). While 12 of 15 replucked mice exhibited recurrent skin lesions (Fig. 2B and C) and 6 had HSV-1 DNA present in the skin by qPCR (Fig. 2D), 0 of 12 shaved mice exhibited recurrent skin lesions, and no HSV-1 was detected in their dermatome skin, indicating that shaving was not a sufficient stimulus to induce reactivation. We next tested whether plucking stimulated reactivation by inducing a systemic response or a local one by plucking the contralateral flank. We infected wild-type mice with HSV-1, and at 35 dpi, we replucked the contralateral flank and shaved the ipsilateral flank to allow recurrent lesions to be observed (Fig. 2A). From eight mice replucked contralaterally, we observed no recurrent dermatome lesions (Fig. 2B and C), and no HSV-1 DNA was detected in the skin (Fig. 2D), indicating that the plucking stimulus had to be at the ipsilateral flank to induce reactivation. Altogether, these data support a model where fur plucking stimulates reactivation of latent HSV-1 from peripheral neurons innervating that site, resulting in recurrent skin lesions.

### Plucking reactivates HSV in multiple strains of laboratory mice

**Fig 3:**
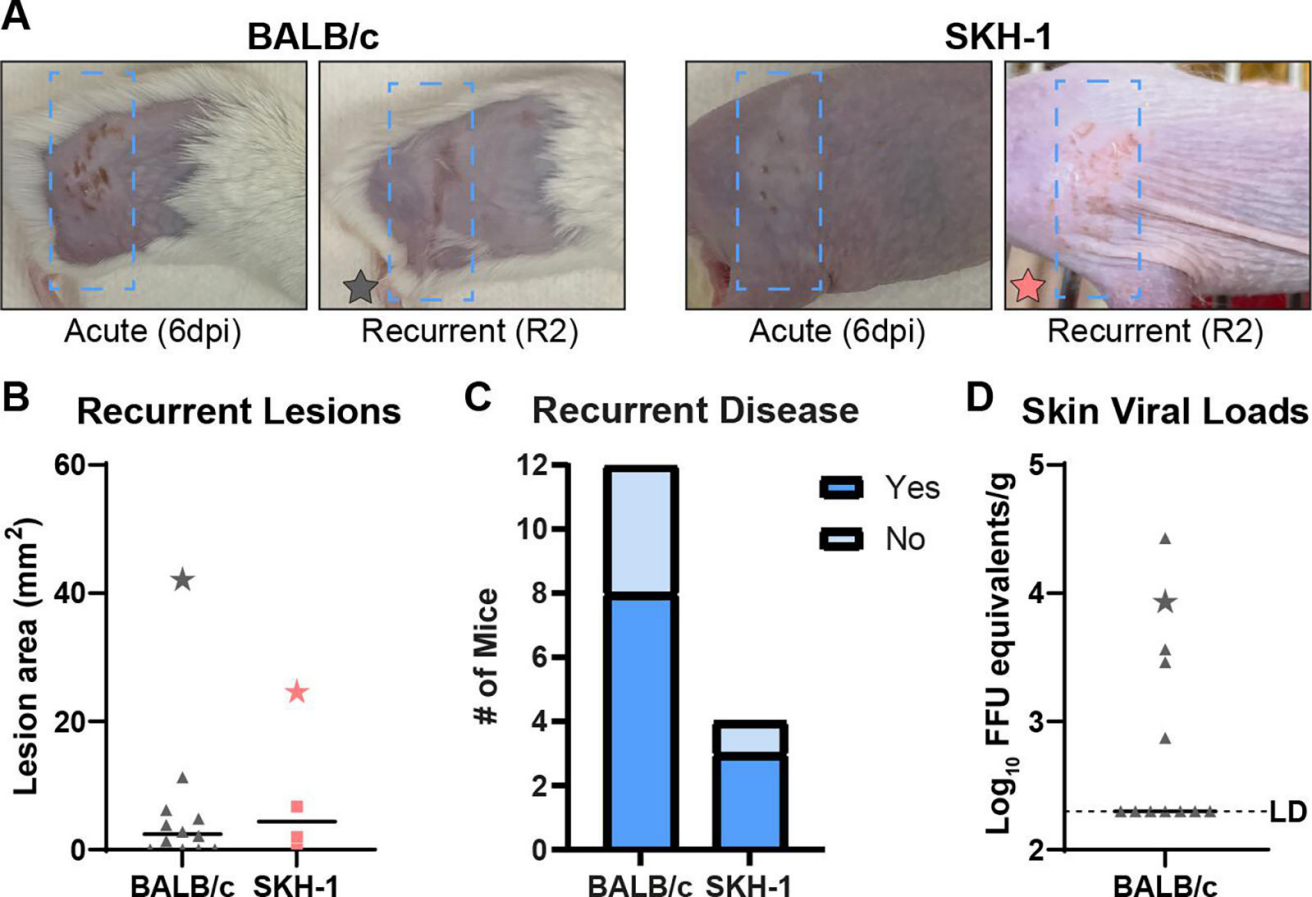
Diverse mouse lines are susceptible to plucking-induced recurrent skin lesions. BALB/c mice and SKH-1 mice were inoculated with 10^5^ or 10^3^ FFU, respectively, of HSV-1 strain NS. After 35 days, BALB/c mice were replucked, and SKH-1 mice were tape stripped to remove fine hairs, and recurrent skin lesions were evaluated. (**A**) Representative serial photos of mice during acute disease (6 dpi) and recurrent disease (R2). Blue boxes denote the dermatome area of interest. (**B**) Skin lesion areas were measured from photographs at R2 using ImageJ. (**C**) Frequency of recurrent disease observed at R2. (**D**) Dermatome skin was harvested from BALB/c mice (*n* = 12) at R2, and viral loads were measured by qPCR. The limit of detection is indicated. Stars indicate the lesion areas and skin viral loads of mice depicted in (**A**).

Having established that fur plucking could reactivate HSV-1 and cause recurrent disease in a C57BL/6 mouse model, we next asked whether this same method would be effective in BALB/c mice, which are commonly used in HSV-1 pathogenesis studies. We infected 12 male BALB/c mice with 10^5^ FFU of HSV-1 strain NS, evaluated acute lesions at 6 dpi, and allowed the lesion to resolve and fur to regrow. We replucked the ipsilateral flank skin 35 dpi (R0) and analyzed lesions 2 days after replucking (R2) (Fig. 3A). Similar to C57BL/6 mice, we observed recurrent lesions in 8 of 12 BALB/c mice (Fig. 3B and C), and we detected HSV-1 DNA in the skin of 5 of 12 BALB/c mice at R2 (Fig. 3D). Notably, all mice with HSV-1 detected in their skin also exhibited a recurrent skin lesion. To further investigate the ability of hair removal to induce HSV-1 reactivation and the breadth of mouse strains susceptible to HSV-1 reactivation by hair removal, we used SKH-1 mice, an immunocompetent mouse line commonly described as “hairless” but which actually have very fine hair (49). We infected SKH-1 mice with 10^3^ FFU of HSV-1 strain NS. Four of nine mice survived the infection (these mice are more susceptible to HSV-1 compared to C57BL/6 mice), and 35 dpi, we removed their ipsilateral flank hair by stripping with autoclave tape. We found that three of four tape-stripped SKH-1 mice developed recurrent lesions by R2 (Fig. 3A through C), indicating that the stimulus of fur removal was sufficient to reactivate HSV-1 from diverse lines of mice.

### HSV-1 and HSV-2 can be reactivated by fur plucking

**Fig 4:**
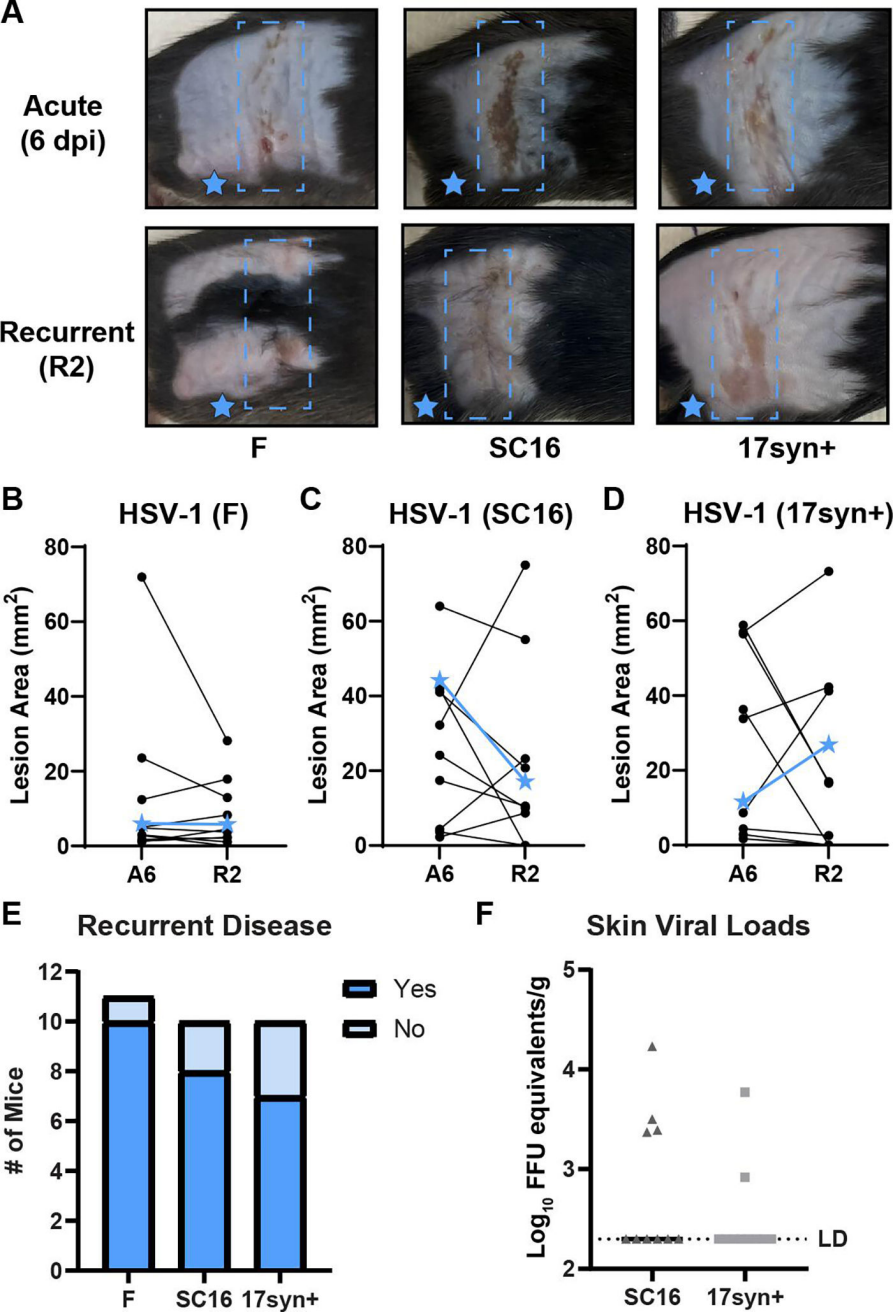
Multiple strains of HSV-1 can be reactivated by fur plucking. WT mice were depilated by plucking 1 day prior to inoculation with 10^5^ FFU of HSV-1 strain F, 10^6^ FFU of HSV-1 strain SC16, or 10^6^ FFU of HSV-1 strain 17syn+. After 50–75 days, mice were replucked on their ipsilateral flank and then evaluated at R2 for disease and viral loads. (**A**) Representative serial photos of the same mouse during acute disease (6 dpi, A6) and recurrent disease (R2). Blue boxes denote the dermatome area of interest. (**B–D**) Paired skin lesion areas were measured using ImageJ from photographs during acute and recurrent disease. Blue stars indicate the lesion areas of the mice depicted in (**A**). (**E**) Frequency of recurrent disease observed at R2. (**F**) Dermatome skin was harvested at R2 from mice infected with HSV-1 strain SC16 and 17syn+, and viral loads were measured by qPCR (*n* = 10 mice per group). The limit of detection is indicated.

**Fig 5:**
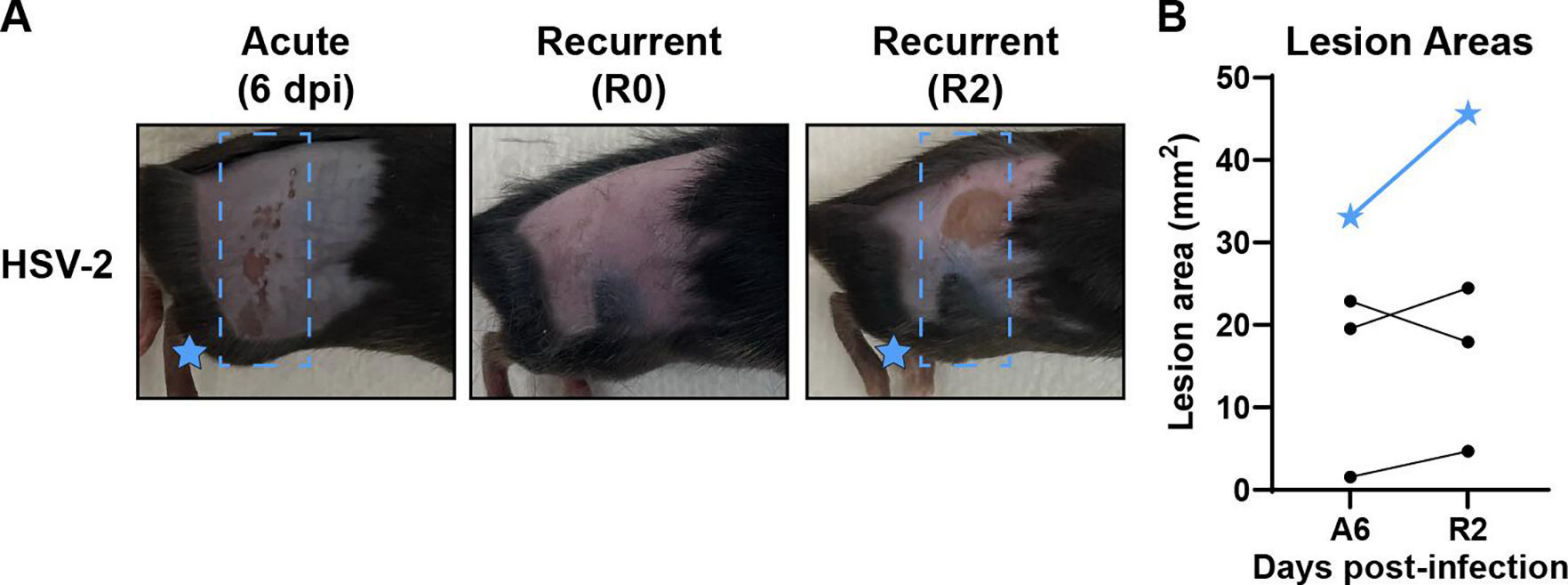
Fur plucking induces HSV-2 recurrent skin disease. WT mice were depilated by plucking 1 day prior to inoculation with 500 FFU of HSV-2 strain 333. After 35 days, mice were replucked and evaluated for recurrent skin disease. (**A**) Representative serial photos of the same mouse during acute disease (6 dpi), immediately post-replucking (R0), and 2 days post-replucking (R2). Blue boxes denote the dermatome area of interest. (**B**) Paired skin lesion areas were measured using ImageJ from photographs during acute and recurrent disease. Blue stars indicate the lesion areas of the mouse depicted in (**A**).

We next asked whether other strains of HSV-1 could be reactivated in the skin by fur plucking, similar to strain NS. We infected C57BL/6 male and female mice with HSV-1 strains F (10^5^ FFU), SC16 (10^6^ FFU), and 17syn+ (10^6^ FFU). We evaluated acute lesions, allowed infection to resolve, and 50–75 days later, we replucked the flank skin and evaluated skin lesions 2 days later (Fig. 4A through E). We found that all three strains were reactivated by fur plucking and exhibited similar rates of recurrence as strain NS: strain F, 11 of 13; strain SC16, 8 of 10; 17syn+, 7 of 10 (Fig. 4E). Additionally, we harvested dermatome skin at R2 from mice infected with SC16 and 17syn+ and quantified skin viral loads by qPCR. We detected viral DNA in the R2 skin from 4 of 10 mice infected with strain SC16 and 2 of 10 mice infected with 17syn+ (Fig. 4F). Furthermore, all mice positive for HSV-1 by qPCR also exhibited a recurrent skin lesion. We next asked whether this reactivation model was specific to HSV-1 or more broadly applicable to HSV-2 as well. We infected wild-type mice with 500 FFU of HSV-2 strain 333 and evaluated lesions 6 dpi. We used a lower dose of HSV-2 than HSV-1 because, at higher doses, most mice succumbed to HSV-2 infection, precluding reactivation studies. At a dose of 500 FFU, 8 of 14 mice exhibited dermatome lesions 6 dpi, and 4 of these survived the infection. We allowed these four mice to recover from the acute infection and regrow their fur, then replucked them 35 dpi. We found 4 of 4 mice developed recurrent lesions after replucking (Fig. 5A and B), and all these mice survived the recurrent infection, consistent with the presence of a protective adaptive immune response at this stage of infection. Altogether, these data show that fur plucking can induce reactivation and recurrent skin disease for both HSV-1 and HSV-2.

### Reactivation by plucking is associated with new viral replication in the skin

Although we detected viral DNA in the skin after reactivation by plucking, we did not detect infectious virus in these samples by titer assays, consistent with prior work in other animal models, showing that viral loads after HSV reactivation are very low (14, 19, 28, 29). To determine whether recurrent lesions were associated with the production of replicating virus, we employed *Stop^f/f^-tdTomato* mice (50) infected with HSV-1 strain SC16 encoding Cre recombinase under the control of a CMV IE promoter (51–53). In this model, Cre recombinase is expressed upon HSV-1 infection, excising a floxed stop codon in host cell DNA and allowing the production of fluorescent tdTomato within the infected cell in a durable manner that is inherited by progeny cells, a model that has been used to identify HSV-infected cells in sensory ganglia (51, 53, 54) and cells that survive infection with arboviruses or respiratory viruses (55–60). We infected male and female *Stop^f/f^-tdTomato* mice with HSV-1 strain SC16-Cre, evaluated acute lesions, and allowed infection to resolve. We replucked the mice 35 days later and either immediately harvested flank skin (R0; *n* = 5) or harvested flank skin 2 days after replucking (R2, *n* = 3), processed skins for histology, and detected HSV-1 antigen by antibody staining and detected tdTomato fluorescence.

**Fig 6:**
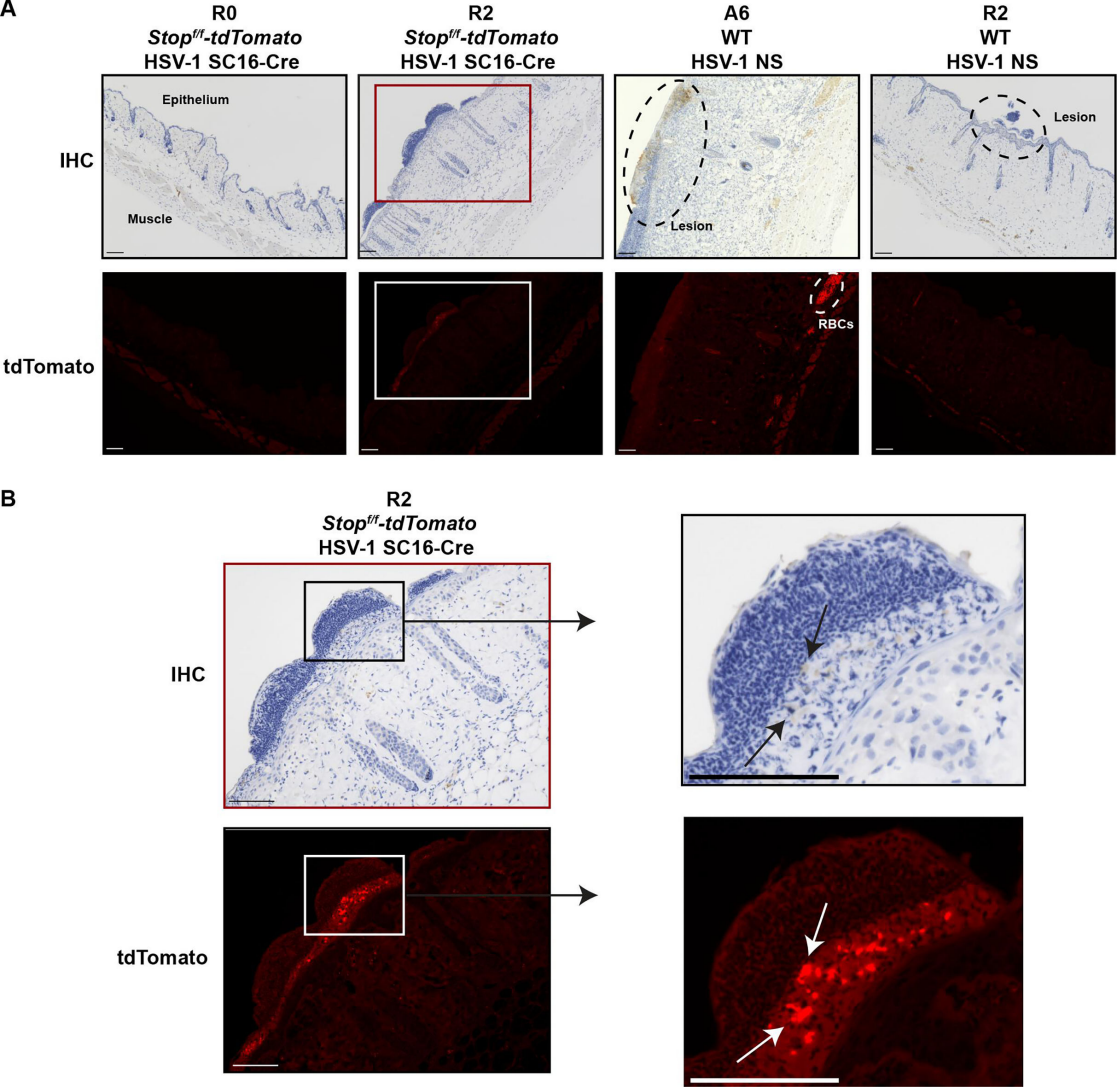
HSV-1 infection in the skin epithelium is evident after fur plucking. *Stop^f/f^-tdTomato* mice were depilated by plucking 1 day prior to inoculation with 10^6^ FFU of HSV-1 expressing Cre recombinase (SC16-Cre). After 35 days, mice were replucked, and skin was harvested for histology at R0 (*n* = 5) or R2 (*n* = 3). WT mice were depilated by plucking 1 day prior to inoculation with 10^6^ FFU of HSV-1 strain NS. Mice were either harvested at A6 (6 dpi) (*n* = 5) or reactivated 35 days later and harvested at R2 (*n* = 3) for skin histology. Slides were processed for IHC with an anti-HSV-1 antibody and imaged in brightfield (IHC) or fluorescent (tdTomato) channels. Images are oriented with epithelium upward and muscle downward. (**A**) Black dashed ovals indicate HSV-1 lesions in the skin epithelium with corresponding immune infiltrate. White oval notes a region of autofluorescence due to red blood cells (RBCs) in the lower dermis. (**B**) Magnified image from panel A (red and white boxes). Black arrows indicate faint HSV-1 antigen staining. White arrows indicate corresponding regions of tdTomato fluorescence in the lesion skin epithelium. Scale bars indicate 100 µM.

We found that HSV-1 antigen staining was faint and sporadic in recurrent lesion skin (Fig. 6A), consistent with our viral load data and studies detecting HSV-1 antigen in mouse corneas following UV-induced reactivation (28, 29). Strikingly, while we observed no tdTomato fluorescence in the skin at R0 (immediately post-replucking), we did observe tdTomato fluorescence in R2 skin lesions (Fig. 6A) and areas with tdTomato signal corresponded to areas with antigen staining (Fig. 6B). In *Stop^f/f^-tdTomato* mice, we did observe red fluorescence in red blood cells and muscle fibers, which are known to exhibit red autofluorescence (61, 62); these cell types were not present in the skin epithelium and did not correspond to HSV antigen staining, supporting the notion that this is an autofluorescent signal, not evidence of HSV infection. Additionally, because we did not observe tdTomato fluorescence in the epithelium of any of our mice at R0 (Fig. 6A), our data suggest that tdTomato fluorescence in the skin epithelium is the result of newly infected cells following reactivation, rather than being epithelial cells that survived, or are derived from survivors, from the acute infection. To rule out the possibility that brown diaminobenzene (DAB) staining exhibited red fluorescence, confounding our interpretations of tdTomato fluorescent signal, we analyzed A6 and R2 skin lesions from wild-type C57BL/6 mice (no tdTomato) infected with HSV-1 strain NS (no Cre recombinase). We observed robust HSV-1 antigen staining during acute infection (A6) and low levels of antigen staining at R2 (Fig. 6A). Notably, we did not observe red fluorescence in the skin epithelium from either A6 or R2 lesions, confirming that DAB signal does not confound detection of tdTomato fluorescence. Altogether, these data support the conclusion that recurrent lesions are associated with HSV-1 infection in the skin, demonstrated by the presence of viral DNA and antigen, albeit at much lower levels than that observed during acute infection.

### Fur plucking can induce multiple recurrent disease episodes in mice

**Fig 7:**
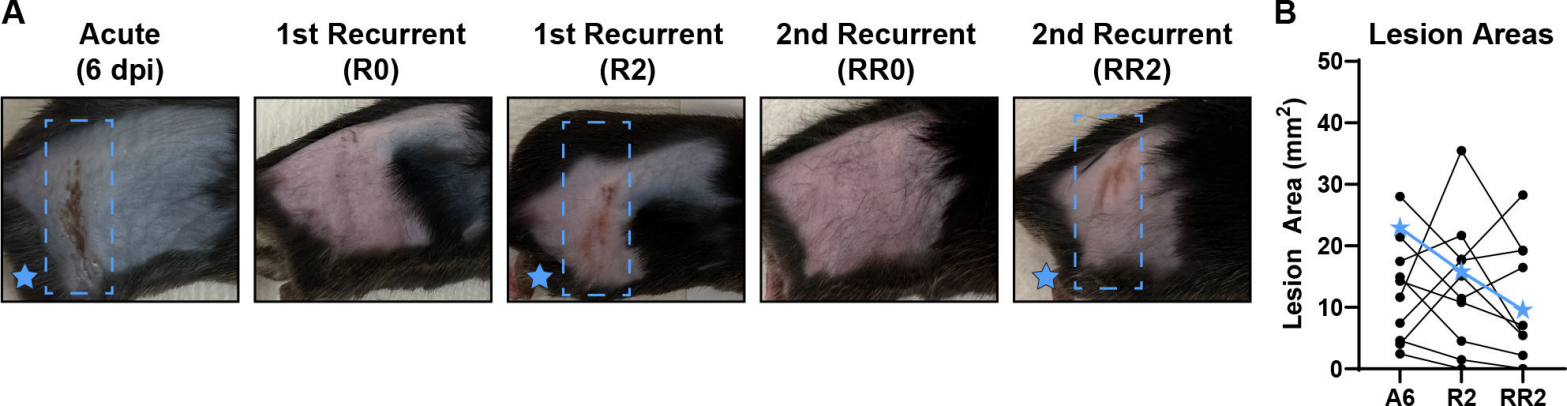
Serial plucking can induce multiple rounds of recurrent HSV-1 disease. WT mice were depilated by plucking 1 day prior to inoculation with 10^6^ FFU of HSV-1 strain NS. After 35 days, mice were replucked, evaluated for recurrent skin disease, and fur was allowed to grow back. Then, 35 days after replucking, mice were replucked again and evaluated for recurrent skin disease. (**A**) Representative serial photos of the same mouse during acute disease (6 dpi, A6), immediately post-replucking (R0), 2 days post-replucking (R2), immediately following the second replucking (RR0), and 2 days after the second replucking (RR2). Blue boxes denote the dermatome area of interest. (**B**) Skin lesion areas for individual mice were measured using ImageJ from photographs at A6, R2, and RR2. Blue stars indicate the lesion areas of the mouse depicted in (**A**).

A defining feature of HSV disease in humans is its ability to cause multiple recurrent disease episodes throughout the lifetime of an infected individual (2). Therefore, we next asked whether fur plucking could stimulate multiple rounds of reactivation and recurrent disease. We infected wild-type mice with 1 × 10^6^ FFU of HSV-1 strain NS and evaluated acute disease 6 dpi. We then replucked the mice 35 dpi, evaluated recurrent skin lesions on R2, and then let the mice recover again and regrow their fur. Then, 35 days after replucking (RR0), we replucked the mice again and evaluated skin lesions. As expected, no skin lesions were evident immediately upon second replucking (RR0), indicating that the prior recurrent skin lesion had cleared. However, after 2 days (RR2), we found that 9 of 11 mice exhibited recurrent skin lesions in the same dermatome as their acute lesions and their first recurrent lesions (Fig. 7A and B). Altogether, these data demonstrate that fur plucking can cause multiple rounds of HSV-1 reactivation in mice.

In summary, we have developed a simple and tractable model to induce HSV-1 and HSV-2 reactivation from latency to cause recurrent skin disease. This new model provides a tractable system for studying pathogenic mechanisms of and therapeutic interventions against HSV reactivation and recurrent disease.

## DISCUSSION

We report a new mouse model to study HSV reactivation and recurrent disease, in which fur plucking is sufficient to stimulate HSV reactivation from latently infected DRG. This new model has several advantages compared to other reactivation models used in the field. The stimulus of fur plucking is fast and easy and requires no specialized equipment. Furthermore, dermatome skin lesions are easy to detect and measure, and the model is applicable to multiple strains of HSV-1 (we tested strains NS, F, SC16, and 17syn+) and HSV-2 (strain 333) and to diverse mouse lines (C57BL/6, BALB/c, and SKH-1). We also disaggregated data by sex and found no differences between male and female mice in their lesion sizes, reactivation rates, or viral loads. Furthermore, we found that plucking is sufficient to induce multiple recurrent episodes in the same mice, allowing for investigations that more closely resemble the recurrent nature of HSV in people. Altogether, the straightforward and tractable nature of this reactivation model makes it well suited to investigating the pathogenic mechanisms of HSV, understanding the immune responses at play during recurrent disease, and evaluating vaccines and therapeutics.

We found that recurrent lesions induced by plucking were associated with new viral infection in the skin. We detected increasing amounts of viral DNA in recurrent skin lesions at R2 and R4 compared to R0 as well as the presence of viral antigen and reporter signal in recurrent skin lesions. The levels of viral DNA and viral antigen detected in recurrent skin lesions were much lower than levels detected during acute infection, which is consistent with the presence of adaptive immune responses generated in response to HSV skin infection (37, 39, 41, 44, 46). Relatively low viral loads in recurrent skin lesions are also consistent with observations in corneal reactivation models, where virus is detected in only a subset of corneas exhibiting recurrent disease (14, 19, 28, 29). Overall, our model is consistent with the understanding that HSV replication is generally well controlled during reactivation and disease is associated with limited viral replication at the site of reactivation (63), suggesting that recurrent disease is predominantly immune mediated.

In the mouse flank model (36–46), the striking dermatome pattern of skin lesions results from the underlying neurobiology of HSV infection, with virus spreading only to areas of skin innervated by infected ganglia (as opposed to spreading laterally across the flank skin); skin lesions are restricted to the dermatome even in *Ifnar1*^−/-^ mice, which develop severe lesions and ultimately succumb to the infection (36). Thus, the observation that plucking-induced recurrent disease occurs in the same dermatome as the acute infection supports a model where recurrent skin lesions result from viral reactivation in sensory ganglia and subsequent spread to innervated areas of skin. However, prior studies have reported HSV reactivation directly from the skin without apparent spread from latently infected neurons (64, 65). We used a Cre-expressing HSV and *Stop^f/f^-tdTomato* mice to permanently label cells previously infected with HSV, a method that has been used to identify cells surviving infection by diverse viruses, including influenza A virus, influenza B virus, SARS-CoV, SARS-CoV-2, Sendai virus, and chikungunya virus (55–60). In this model, we observed no tdTomato fluorescence immediately following replucking (only 2 days later, concurrent with recurrent skin lesions), altogether arguing against latently infected cells in the skin being the source of reactivating virus.

Our observation that fur plucking the ipsilateral flank is sufficient to induce reactivation, whereas shaving or contralateral flank plucking did not induce reactivation, suggests that fur plucking triggers a response in innervating peripheral neurons and provides an opportunity to study the neurobiology of HSV infection. Cell culture models of HSV latency in cultured neurons have defined stimuli, such as axon damage, growth factor deprivation, and inflammatory cytokines, as inducing the epigenetic changes required to activate viral gene expression from the latent genome (13, 66–69). Hair follicles are encircled by mechanosensory neurons, which we posit are damaged during reactivation by plucking. Future studies will investigate the mechanism by which plucking modulates peripheral neurons to induce reactivation and recurrent disease and will investigate the effects of other stimuli in this new mouse model of HSV reactivation.

## MATERIALS AND METHODS

### Viruses and cells

Virus stocks were grown in Vero (African green monkey kidney epithelial) cells in 850 cm^2^ roller bottles. Supernatants and cells were then collected and were put through two freeze-thaw cycles before spinning down cell debris at 3,000 rpm for 30 min at 4°C. Supernatants were then concentrated by ultracentrifugation at 25,000 rpm (SW32 Ti rotor; Beckman Coulter #369650) for 1.5 hours and subsequently resuspended in cold PBS. Vero cells were maintained in Dulbecco’s modified Eagle medium containing 5% heat-inactivated fetal bovine serum (FBS) and L-glutamine at 37°C with 5% CO_2_ containing 2% FBS, L-glutamine, and HEPES at 37°C with 5% CO_2_. HSV-1 strain NS was obtained from Dr. Harvey Friedman (University of Pennsylvania) (70). HSV-1 strain 17syn+ was obtained from Dr. David Leib (Dartmouth University). HSV-1 strain F was obtained from Dr. Bin He (University of Illinois Chicago). HSV-1 strains SC16 and SC16 CMV-Cre were obtained from Dr. Thomas Kristie (NIAID) (52) and were originally from Dr. Stacy Efstathiou (Cambridge University) (51). HSV-2 strain 333 was obtained from Dr. Steven Bachenheimer (UNC). Virus stock titer was quantified by focus-forming assay on Vero cells (36). In brief, viral stocks were serially diluted and inoculated onto Vero cell monolayers with a methylcellulose overlay. Twenty hours after inoculation, wells were fixed by 4% PFA for 1 hour at room temperature and stained to detect viral foci. Viral foci were detected using 1:10,000 dilution of αHSV rabbit antibody (Dako #B0114) and 1:50,000 dilution of goat anti-rabbit HRP-conjugated antibody (Sigma #12–348), and TrueBlue peroxidase substrate (KPL). Antibody incubations were performed for at least 1 hour at room temperature. Foci were counted on a CTL Immunospot analyzer.

### Mice

Experiments used 8–12-week-old male and female mice.

C57BL/6 mice were purchased from JAX (#664) and bred in house. BALB/c mice were purchased from JAX (#651). SKH-1 mice (Charles River strain #477) were received from Dr. Brian Conlon (UNC). *Stop^f/f^-tdTomato* mice (Ai14, JAX #7914) (50) were obtained from Dr. Karl Shpargel (UNC) and bred in-house.

### HSV skin infections

One day prior to infection, mice were anesthetized by nose-cone isoflurane and depilated by manual plucking (using fingers) on the right flank unless otherwise indicated. One day later, mice were anesthetized by chamber isoflurane for infections. To perform infections, we abraded the skin of anesthetized, depilated mice with ~10 closely spaced punctures (~5 mm^2^ total area) using a Quintip allergy needle (Hollister Stier #8400ZA). Immediately after abrasion, we overlaid 10 µL of viral inoculum (virus + 1% FBS in PBS) and allowed the inoculum to dry while mice were anesthetized. For reactivation, mice were anesthetized by nose-cone isoflurane and depilated by plucking on the right flank. Shaving was performed using Wahl Arco professional animal clippers (#8786 100). SKH-1 mice were tape stripped using autoclave tape across their right flank.

### Viral genome quantification

The entire dermatome region of skin was harvested, collecting the same total area of skin from each mouse regardless of lesion size. Skin was homogenized in 500 µL of PBS with silica beads on a MagNAlyser instrument (Roche). DNA was then extracted from 200 µL of homogenate using the DNeasy Blood and Tissue Kit (Qiagen #69504). Extracted HSV-1 genomes were then quantified by TaqMan qPCR on a CFX96 Touch real-time PCR detection system (Bio-Rad) against a standard curve generated by serial dilutions of DNA extracted from an HSV-1 stock at 10^8^ FFU/mL. HSV-1 genomes were detected using the following primers against the UL23 gene: F primer 5′-TTGTCTCCTTCCGTGTTTCAGTT-3′, R primer 5′-GGCTCCATACCGACGATCTG-3′, and probe 5′-FAM-CCATCTCCCGGGCAAACGTGC-MGB-NFQ-3′ (54).

### Lesion area quantification

Skin lesion area was quantified as previously described (36). Mice were anesthetized and photographed using an iPhone camera next to a ruler and an identifying card. Thereafter, images were analyzed using Image J in which pixels were converted to millimeters using the reference ruler, and then lesions were outlined using the freehand tool, and calculated area within the freehand designations was reported.

### Histology

Skin was prepared for histology at the indicated time points by collecting the entire depilated region of the right flank (lesional and healthy skin), flattening the skin on cardstock, and submerging in 10% neutral buffered formalin for 48 hours at 4°C. After fixation, skins were washed and resuspended in 70% ethanol and stored at 4°C until submission to the UNC Pathology Services Core Facility. To prepare fixed skin for submission, two cuts of skin were taken: (i) a horizontal section through the flank to capture lesion and healthy skin and (ii) a vertical cut to capture the diseased dermatome region of skin. Thereafter, samples were stored in 70% ethanol and processed by the core. Slides were stained for HSV-1 (Dako #B0114, 1:1500), imaged on a Keyence BZ-X810 fluorescence microscope, and analyzed using BZ-X800 software.
